# Association of Premature Immune Aging and Cytomegalovirus After Solid Organ Transplant

**DOI:** 10.3389/fimmu.2021.661551

**Published:** 2021-05-27

**Authors:** Lauren E. Higdon, Claire E. Gustafson, Xuhuai Ji, Malaya K. Sahoo, Benjamin A. Pinsky, Kenneth B. Margulies, Holden T. Maecker, Jorg Goronzy, Jonathan S. Maltzman

**Affiliations:** ^1^ Department of Medicine/Nephrology, Stanford University, Palo Alto, CA, United States; ^2^ Department of Medicine/Immunology & Rheumatology, Stanford University, Palo Alto, CA, United States; ^3^ Human Immune Monitoring Center, Stanford University, Palo Alto, CA, United States; ^4^ Department of Pathology, Stanford University, Palo Alto, CA, United States; ^5^ Department of Medicine/Infectious Diseases and Geographic Medicine, Stanford University, Palo Alto, CA, United States; ^6^ Cardiovascular Institute, Perelman School of Medicine, University of Pennsylvania, Philadelphia, PA, United States; ^7^ Department of Microbiology & Immunology, Stanford University, Palo Alto, CA, United States; ^8^ Department of Medicine, VA Palo Alto Health Care System, Palo Alto, CA, United States

**Keywords:** immunosenescence, cytomegalovirus (CMV), flow cytometry, Telomere, transplantation immunobiology

## Abstract

Immune function is altered with increasing age. Infection with cytomegalovirus (CMV) accelerates age-related immunological changes resulting in expanded oligoclonal memory CD8 T cell populations with impaired proliferation, signaling, and cytokine production. As a consequence, elderly CMV seropositive (CMV^+^) individuals have increased mortality and impaired responses to other infections in comparison to seronegative (CMV^–^) individuals of the same age. CMV is also a significant complication after organ transplantation, and recent studies have shown that CMV-associated expansion of memory T cells is accelerated after transplantation. Thus, we investigated whether immune aging is accelerated post-transplant, using a combination of telomere length, flow cytometry phenotyping, and single cell RNA sequencing. Telomere length decreased slightly in the first year after transplantation in a subset of both CMV^+^ and CMV^–^ recipients with a strong concordance between CD57^+^ cells and short telomeres. Phenotypically aged cells increased post-transplant specifically in CMV^+^ recipients, and clonally expanded T cells were enriched for terminally differentiated cells post-transplant. Overall, these findings demonstrate a pattern of accelerated aging of the CD8 T cell compartment in CMV^+^ transplant recipients.

## Introduction

Immune aging has significant impacts on immune function. Signaling, phagocytosis, and antigen presentation by cells of the innate immune system become impaired over the course of a lifetime [reviewed in (1)]. B and naïve T cell populations are reduced in size, with altered phenotypes (2, 3). Memory lymphocytes and other differentiated cells become immunosenescent, characterized by shortened telomeres and impaired functionality, including class-switch in B cells as well as TCR signaling and cytokine production in T cells (2, 3). These alterations impair responses to vaccination and infection in the elderly.

A major contributor to immune aging is cytomegalovirus (CMV), a common human herpesvirus infection. Primary infection with CMV is rapidly controlled but never cleared, with virus remaining as a latent infection (4). In response to reactivation with expression of specific CMV proteins, a portion of the CD8 memory repertoire gradually expands over time, in a process termed memory inflation (5–7). Memory inflation can often induce an immunosenescent phenotype. Inflated T cells are oligoclonally expanded with characteristics of impaired proliferation, shortened telomeres, reduced telomerase activity, altered cytokine production, and a terminally differentiated phenotype (8, 9). Moreover, these cells can comprise up to 40% of the T cell repertoire in the aged (10).

The combination of an increased proportion of CD8 T cells and CMV seropositivity has been shown to represent an immune risk profile (IRP) associated with increased risk of mortality within a 2 year period in the very old (11, 12). The IRP is defined as gain of CD8 T cells, loss of CD4 T cells and B cells, and poor T cell proliferation (13). CD8 T cell expansion in the IRP is linked to CMV and represents an increase in highly differentiated CD28^–^ and CD57^+^ CD8 T cells (12). Risk of cardiovascular mortality in octogenarians is increased by both CMV seropositivity and immunosenescent T cells (14). Chronic and end-stage kidney disease contribute to T cell immunosenescence (15, 16); this process is further enhanced by CMV (17). In addition, CMV impairs the ability to respond to other antigen challenge in the aged (7).

CMV is a major opportunistic infection in transplant recipients and other immunosuppressed or immunocompromised individuals (18–20). Whether CMV-induced immune aging contributes to post-transplant morbidities remains unknown. Three recent studies have demonstrated that memory inflation may occur at an accelerated rate after transplantation (21–23). A fourth found expansion of terminally differentiated CD8 T cells within a year post-transplant specifically in patients with detected CMV viremia (24).

To determine whether the observed accelerated inflation after transplantation is associated with increased immune aging, we analyzed immunosenescence in recipients of heart or kidney transplantation. We found that telomere length decreases slightly in the first year after transplantation and demonstrated that CD57 can be used as a proxy for aging based on flow cytometry-based telomere length assessment. We further demonstrated that the proportion of CD8 T cells with hallmarks of an aged/immunosenescent phenotype increases post-transplant specifically in CMV seropositive (CMV^+^) recipients, and that this shift results from the accumulation of cells of a CD57^+^ highly differentiated phenotype.

## Materials and Methods

### Human Subjects

**Figure 1:**
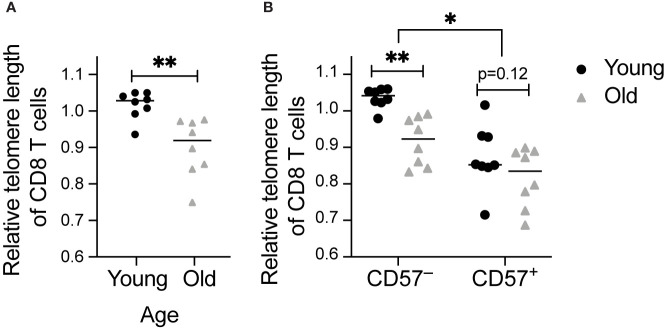
Telomere length decreases with age and in CD57^+^ CD8 T cells: Telomere length was calculated by subtraction of value without telomere probe added (**Supplementary Figure 1C**) and normalization to value for live cells from a healthy volunteer. Measurements were completed in CD8 T cells from young (18-35 years old) and old (60-80+ years old) healthy individuals. Comparison of telomere length of **(A)** total and **(B)** CD57^+^ and CD57^–^ CD8 T cells between age groups. Dots represent individual samples and lines represent medians. Statistics were **(A)** Mann-Whitney test or **(B)** two-way ANOVA. *p < 0.05, **p < 0.01. n = 8 per group.

Recipients of heart or kidney transplant were enrolled at the University of Pennsylvania, Stanford University or the VA Palo Alto Health Care System either pre-transplant or within one year of transplant. All patients were treated per standard of care at the treating institution, which included similar three drug immunosuppressive therapy with prednisone, tacrolimus, and mycophenolate for all patients. Induction therapy varied depending on transplanted organ and center. There were 18 enrolled kidney recipients, who received induction therapy of either rabbit anti-thymocyte globulin (rATG, 15 subjects) or basiliximab (3 subjects). There were 10 enrolled heart recipients, who received induction therapy of basiliximab (7 subjects), rATG (1 subject) or no induction (2 subjects). Blood samples were collected pre-transplant and at intervals varying between 3 and 12 months after transplant up until 2 years post-transplant. If data for a time point are omitted, that sample was not available for that individual. All patients and organ donors were tested pre-transplant for CMV serostatus, and this information is included in all analyses (**Table 1**). CMV viral load was monitored per clinical protocols or indication. Additional testing of samples from subject 14 was conducted on sera at the Stanford Clinical Virology lab using the *artus* CMV MDx Rotor-Gene kit (Qiagen, Germantown, MD) targeting the CMV major immediate-early (MIE) gene. This test has a lower limit of quantification at 135 IU/mL of serum. This study was approved by the IRBs at the University of Pennsylvania (protocol number 817637) as well as the Veterans Administration Palo Alto Health Care System and Stanford University (protocol number 38882). Identifiable source information was blinded to those completing studies. Healthy volunteer samples were collected from the University of Pennsylvania Human Immunology Core, Stanford University (protocol number 17984), or the Stanford Blood Center. The healthy volunteer samples used in **Figure 1** were a cohort of 8 young (aged 18-35) and old (aged 60-80^+^) individuals. These studies were in accordance with the Declaration of Helsinki and all participants gave written informed consent prior study inclusion.

**Table 1:** Clinical information on each subject.

#	Sex	Age decade at transplant	Organ	Donor CMV	Recipient CMV	Induction therapy	Valganciclovir duration	CMV PCR
1	M	60s	Kidney	+	+	rATG	3 months	N.D.
2	M	60s	Heart	+	+	rATG	1 year	N.D.
3	M	30s	Kidney	–	–	rATG	2 years	N.D.
4	M	50s	Kidney	–	+	rATG	150 days	N.D.
5	M	60s	Kidney	–	+	Basiliximab	1 month	N/A
6	M	60s	Kidney	+	+	Basiliximab	N/A	N/A
7	M	70s	Kidney	+	–	Basiliximab	150 days	N.D.
8	F	60s	Heart	–	+	Basiliximab	3 months	N.D.
9	M	60s	Heart	–	+	N/A	3 months	N.D.
10	M	50s	Kidney	–	–	rATG	3 months	N.D.
11	M	70s	Kidney	+	–	rATG	6 months	N.D.
12	M	60s	Kidney	–	–	rATG	3 months	N/A
13	M	50s	Kidney	–	–	rATG	3 months	N/A
14	F	50s	Heart	+	+	Basiliximab	See **Figure 5A**	
15	F	60s	Kidney	+	+	rATG	3 months	N.D.
16	M	30s	Heart	–	+	Basiliximab	5 months	N/A
17	F	50s	Heart	–	+	Basiliximab	1 month	N.D.
18	M	60s	Heart	–	+	Basiliximab	4 months	N.D.
19	F	40s	Kidney	–	+	rATG	4 months	N.D.
20	M	40s	Kidney	–	+	rATG	1 month	N.D.
21	F	50s	Kidney	–	+	rATG	4 months	N.D.
22	M	40s	Kidney	+	+	rATG	5 months	N/A
23	M	40s	Kidney	–	+	rATG	3 months	N/A
24	M	50s	Kidney	–	+	rATG	3 months	N.D.
25	M	60s	Kidney	+	+	rATG	3 months	N.D.
26	M	50s	Heart	–	+	N/A	3 months	N/A
27	M	50s	Heart	+	+	Basiliximab	3 months	N/A
28	M	40s	Heart	–	–	Basiliximab	N/A	N/A

**Figure 2:**
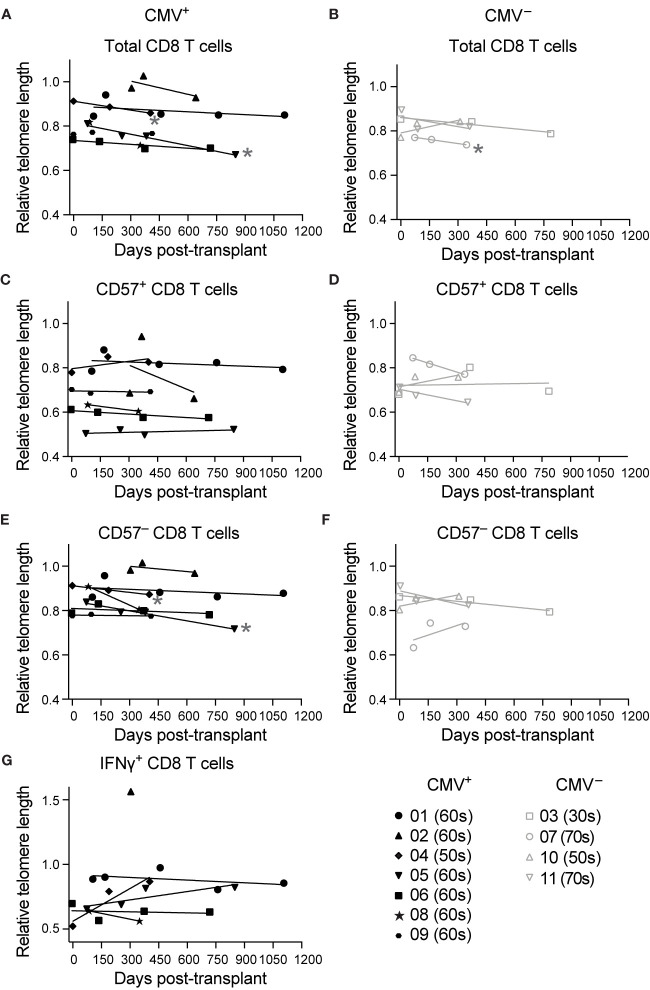
Telomere length decreases in total CD8 T cells post-transplant in a subset of subjects: Samples were stimulated for 6 hrs with IE-1 and pp65. Relative telomere length was calculated as described in the legend of **Figure 1**. Values were calculated for **(A, B)** total CD8 T cells, **(C, D)** CD57^+^ CD8 T cells, **(E, F)** CD57^–^ CD8 T cells, and **(G)** IFNγ^+^ CD8 T cells for **(A, C, E, G)** CMV^+^ and **(B, D, F)** CMV^–^ recipients. Dots represent individual samples and lines represent simple linear regressions. In the key at lower right, number indicates subject ID, and parenthetical number indicates subject age decade on day of transplant. * indicates statistically significant slope (p<0.05) of indicated regression. n = 7 (CMV^+^) or 4 (CMV^–^) subjects.

**Figure 3:**
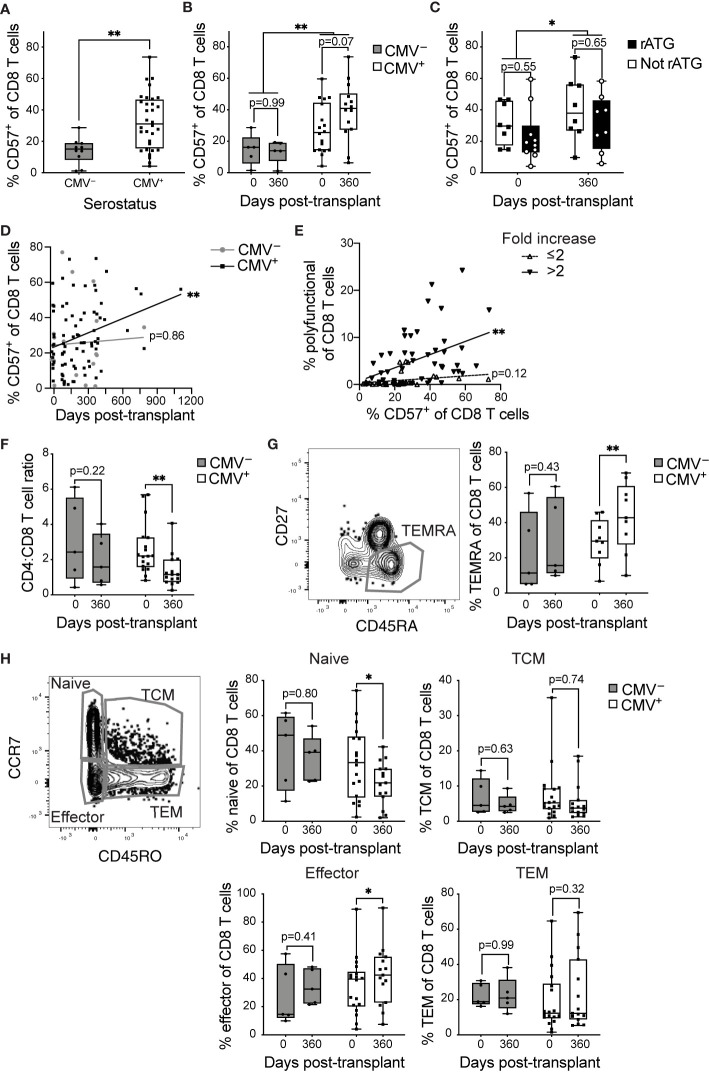
CD8 T cells become phenotypically aged in CMV^+^ transplant recipients: T cells from CMV^+^ and CMV^–^ transplant recipients were analyzed by flow cytometry. % CD57^+^ was measured at **(A)** combined time points **(B)** pre-transplant *versus* one year post-transplant. **(C)** % CD57^+^ of CD8 T cells pre-transplant *versus* one year post-transplant with CMV^+^ recipients grouped based on induction therapy. **(D)** % CD57^+^ of CD8 T cells plotted *versus* time. **(E)** % CD57^+^ plotted *versus* % polyfunctional (a measure of memory inflation) for CMV^+^ recipients. Polyfunctionality was defined with Boolean gating as in **Supplementary Figure 2B** and (21). The data are divided based on the magnitude of inflation, as fold change >2 or ≤2. **(F)** Ratio of proportion of CD4 to CD8 T cells. **(G)** Representative gating of CD45RA^+^CD27^–^ TEMRA (left). TEMRA population was defined as CD45RA^+^CD27^–^ or CD45RA^+^CCR7^–^CD45RO^–^ and proportion TEMRA of CD8 T cells determined (right). **(H)** Naive, memory, and effector populations defined based on CCR7 and CD45RO (left). Proportion of CD8 T cells belonging to naive (top middle), TCM (top right), effector (bottom middle), and TEM (bottom right) determined. **(A–C, F–H)** Dots represent individual samples and box and whisker plots represent the range from minimum to maximum. Statistics were **(A)** Mann-Whitney test, **(B, C, F–H)** mixed-effects analysis with Sidak correction for multiple comparisons, **(D, E)** simple linear regression (lines on graph) with p-values displayed at right. *p<0.05, **p<0.01. **(A, B, F, H)** n = 5-18 per group, **(C)** n= 8-10 per group, **(D)** n= 7-20 per group, **(E)** n = 20 **(G)** n= 5-9 per group.

**Figure 4:**
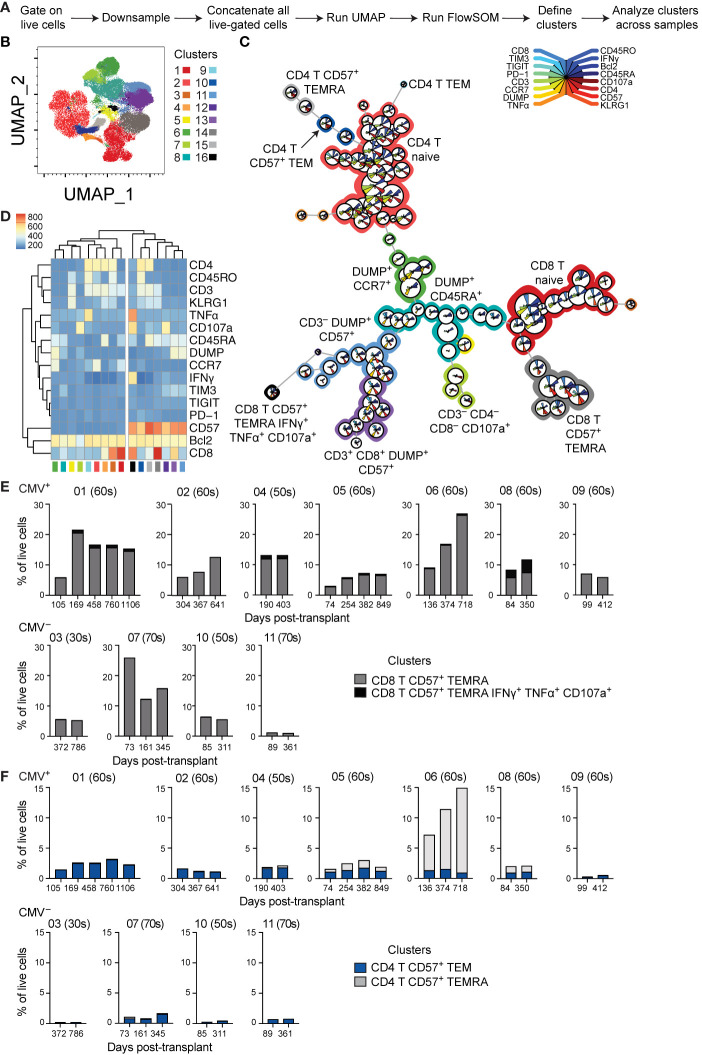
Unbiased analysis identifies expansion of aged CD8 T cell population post-transplant: **(A)** Live-gated flow cytometric phenotypic data from eleven transplant recipients were downsampled to 11,500 cells each and compensated data concatenated. **(B)** Dimensions were reduced using UMAP. **(C)** FlowSOM was run to cluster the data and identify populations. **(D)** Heatmap generated by FlowSOM plug-in defines gene expression of each FlowSOM cluster. Clustering on the heatmap was unsupervised. The percentage of live cells represented by each CD57^+^ **(E)** CD8^+^ or **(F)** CD4^+^ cluster was calculated and graphed, with individual patients separated. **(E, F)** The number above each graph is the subject ID, and the parenthetical number the indicates subject age decade on day of transplant. CMV^+^ subjects listed on top, and CMV^–^ subjects listed on bottom. n= 7 (CMV^+^) or 4 (CMV^–^) subjects.

**Figure 5:**
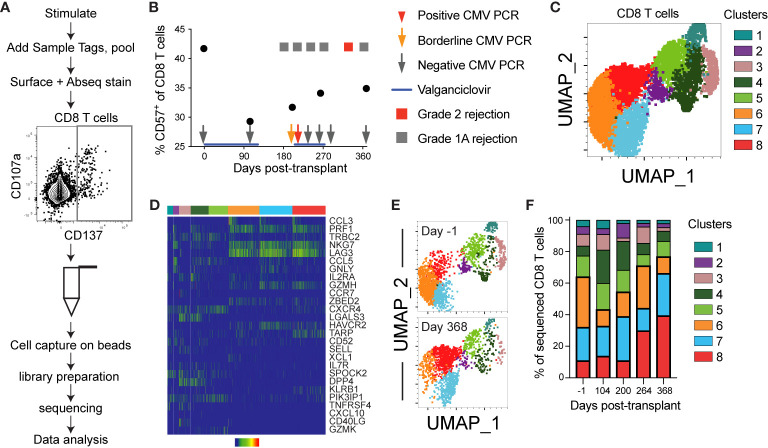
Single cell sequencing reveals post-transplant CD8 T cell aging: CD8 T cells from one CMV^+^ heart transplant recipient (subject 14) from five time points encompassing pre- to one year post-transplant were sequenced using the BD Rhapsody system to detect V(D)J complementarity determining region (CDR)3, T cell associated gene expression, and surface expression of oligo-conjugated CD45RA. **(A)** Schematic of the experimental workflow, including sort gate for CD137^+^ IE-1-responsive T cells. **(B)** The percentage CD57^+^ of CD8 T cells was calculated from analysis of surface stain data from this subject from a prior experiment on aliquots of the same samples. Overlaid onto the graph are information about her course of antiviral prophylaxis (valganciclovir), CMV PCR testing, and rejection episodes. The positive CMV PCR (red arrow) was 213 international units/mL and the borderline CMV PCR (orange arrow) was detectable but below the 135 IU/mL limit of quantitation. The grade 1A rejection events were not treated, and the grade 2 rejection was treated by steroid. **(C)** The Seurat plug-in in SeqGeq was used to cluster all CD8 T cell data from this subject and project the clusters onto UMAP. **(D)** The three genes with the highest and lowest fold change in each cluster were determined, and a heatmap of expression levels created in SeqGeq. The order of genes in the heatmap is determined by expression level, with the most highly expressed genes on top. In some cases, the same gene was in the top or bottom three for multiple clusters, so the total number does not reflect six per cluster. Each bar in the heatmap represents an individual cell. **(E)** UMAP overlay of clusters at the first (day -1) and final (day 368) time points. **(F)** Frequencies represented by each cluster at each time point. n=1 subject.

**Figure 6:**
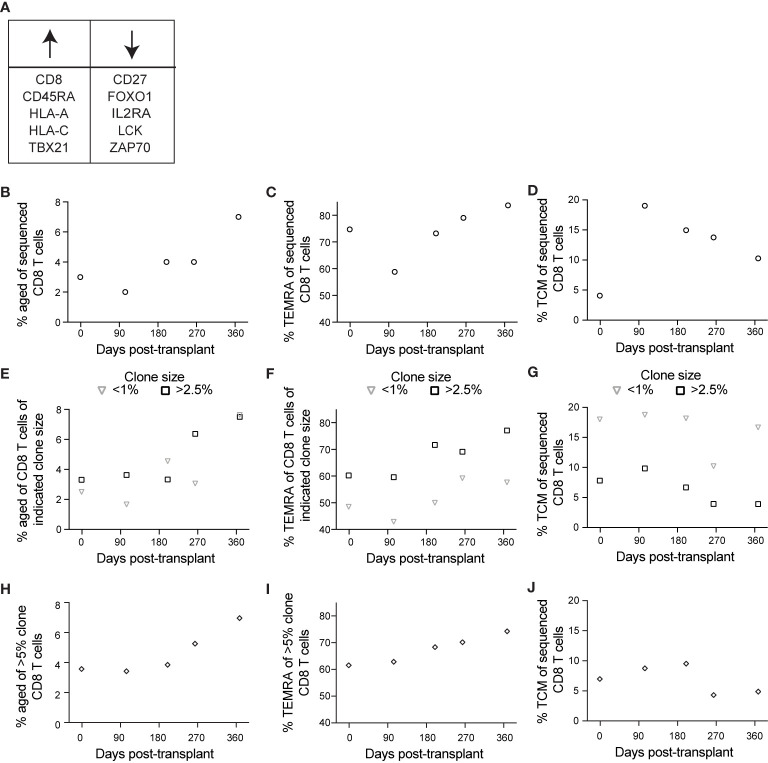
Single cell sequencing reveals an association between aging and CD8 T cell clonality: BD Rhapsody analysis of subject 14 was completed as described in the legend to **Figure 5**. **(A)** We identified aged cells in the sequencing data using sets of genes positively and negatively associated with immune aging. **(B)** Based on this definition, we determined frequency aged of CD8 T cells at each time point. **(C)** We determined percentage TEMRA, with TEMRA defined as CD45RA^+^CD27^–^CCR7^–^. **(D)** We determined percentage TCM, with TCM defined as CD45RA^–^CCR7^+^. We then calculated percentage **(E)** aged, **(F)** TEMRA, and **(G)** TCM of CD8 T cells of indicated clone sizes, with <1% representing small clones and >2.5% representing large clones. To specifically analyze the largest clones, we then calculated percentage **(H)** aged, **(I)** TEMRA, and **(J)** TCM of those clones represented by >5% of CD8 T cells at one or more time points, representing the largest clones. n=1 subject.

In Induction column, N/A means no induction therapy. In Valganciclovir column, N/A means no antiviral prophylaxis. In CMV PCR column, N/A means the PCR was not completed. N.D. means CMV DNA was not detected. This cohort overlaps with that published in (21), but the subject ID is distinct. Subjects 01-11 were included in the analyses in **Figures 2** and **4**. Subjects 01-13 and 15-28 were included in the analyses in **Figure 3**. Subject 14 was included in the BD Rhapsody analysis in **Figures 5** and **6**.

### Blood Collection and Processing

Blood was collected and processed as described (25). Briefly, blood was collected in vacutainers containing EDTA (Ethylenediaminetetraacetic acid) as anticoagulant and processed within 1-24 hours using a Ficoll gradient. The type of anticoagulation used during phlebotomy may impact downstream assays (26). We selected EDTA for all transplant recipient samples, which has been shown not to impact cytokine production in T cells after Ficoll purification (27). Healthy volunteer blood samples from young/old individuals used in **Figure 1** were isolated in leukapheresis chambers with a negligible amount of citrate-based anticoagulant, or in vacutainers containing sodium heparin. Neither of these anticoagulants inhibit cytokine production. All assays including cytokine production were conducted on samples prepared in EDTA. Peripheral blood mononuclear cells (PBMC) were frozen at 5-10x10^6^ cells/mL in fetal bovine serum (FBS, Gemini) with 10% dimethyl sulfoxide (DMSO, Sigma).

### Peptide Libraries

Cells were stimulated with peptide libraries for the immunodominant CMV polypeptides immediate early-1 (IE-1) and phosphoprotein 65 (pp65, GenScript, Piscataway, NJ) consisting of 15 amino acid peptides with 11 amino acid overlap for the length of the polypeptides (28).

### Cell Stimulation

Samples were thawed as described (25) in batches of all time points for one patient along with a healthy volunteer sample. In some cases, multiple patients were batched together. For the analysis of old and young healthy volunteer samples, all were thawed and analyzed together. Cells were rested overnight in R10 [RPMI with 10% FBS, 1% Penicillin/Streptomycin (Thermo Fisher, Waltham, MA), and 1% L-glutamine (Thermo Fisher)] with 10 units/mL DNase (Roche Life Science, Indianapolis, IN). Cells were re-suspended at 2x10^6^/mL in fresh R10 with 10 units/mL DNase and 3 μL/mL CD28/CD49d costimulatory reagent (BD Biosciences, San Jose, CA), and split between stimulated and unstimulated conditions. For phenotyping, cells were stimulated with IE-1 (0.8 μg/mL) or pp65 (3.2 μg/mL) peptide libraries. For subsequent analysis of telomere length, cells were stimulated with IE-1 and pp65 libraries together (0.8 μg/mL, 2.4 µg/mL). When used in the stain, antibody to human CD107a (BioLegend, San Diego, CA) was added at this time. After the first hour,brefeldin A (2 mg/mL; Life Technologies), monensin (BD Golgistop at 0.7 µL/mL) were added. Stimulation occurred in an incubator at 37°C and 5% CO_2_ at an 80° angle for six hours and was stopped by addition of 3 mL cold PBS (Thermo Fisher). Cells were detached from tubes *via* incubation for 10 min in PBS with 2 mM EDTA (Thermo Fisher) at 37°C. While there was an unstimulated control for all samples, the data presented in the figures are all from stimulated samples.

### Stain for Flow Cytometric Phenotyping

Following stimulation as described in Cell Stimulation section, cells were first stained in an antibody to human C-C chemokine receptor (CCR)7 (BioLegend) diluted at 1:20 in PBS for 30 min at 37°C and then in Zombie Aqua dye (BioLegend) diluted at 1:400 in PBS for 20 min at room temperature. Cells were washed and spun down at 1500 rpm for 5 min, resuspended in surface stain prepared in Brilliant Stain Buffer (BD Biosciences), incubated for 30 min at room temperature, and then washed and spun down. Surface stain included an Fc receptor, CD16, for blocking. Cells were fixed using the BD Cytofix/Cytoperm kit and protocol. Fixed cells were stained for 1 h at room temperature with intracellular antibodies diluted in 1X Perm/Wash buffer from the kit. Cells were washed and spun down and then resuspended in PBS with 1% paraformaldehyde (Alfa Aesar, Haverhill, MA). Before samples were run on a flow cytometer, they were resuspended in PBS.

Antibodies conjugated to fluorescein, phycoerythrin (PE) or PE conjugates, allophycocyanin (APC) or APC conjugates, Alexa Fluor 700, Brilliant Violet dyes 421, 570, 605, 650, 711, or 785, and Brilliant Ultraviolet dyes 395, 496, and 737 were purchased from BioLegend, eBioscience, BD, Life Technologies, Abcam (Cambridge, UK), or Beckman-Coulter (Pasadena, CA). Full stain panels for every figure are listed in **Supplementary Table 1**.

### Stain for Telomere Length Analysis

Following stimulation as described in Cell Stimulation section, PBMC were first stained with Zombie Aqua and then with antibody to human CD45 (clone HI30, BioLegend or BD Biosciences) or β2M (clone 2M2, BioLegend). These antibodies were used in multiple fluorochromes for the purpose of barcoding. Samples from a single healthy volunteer were included in every mix as a control to normalize telomere length measurements. After this step, cells were washed, spun down, and combined into tubes of up to 9 fully barcoded samples (**Supplementary Figure 1A**). Cells were then stained as described in the Stain for Flow Cytometric Phenotyping section, except in PBS containing 0.5% BSA and 2mM EDTA for the surface stain step. Stain panel is in **Supplementary Table 1A**. Prior to fixation, HeLa cells (ATCC CCL-2) were added at a 1:4 ratio relative to the number of PBMC. HeLa cells are a high telomere content control, and telomere length of HeLa cells was controlled by freshly thawing aliquots from the same passage number on the day of each experiment. After fixation, cells were resuspended in 250 µL PBS with 1% FBS and treated with chemical cross-linker bissulfosuccinimidyl suberate at a final concentration of 5 mM on ice for 30 minutes per a previously published protocol (29). The reaction was quenched with a 20-minute incubation in quenching solution (PBS with 100 mM Tris-HCl, 50 mM NaCl). Samples were transferred to Eppendorf tubes and stained for telomere length using the Dako Telomere PNA kit/FITC (Agilent) and protocol. In figures, all data for telomere length are from stimulated cells.

### Compensation Controls

Compensation controls were prepared by using unstained and single stained cells, eBioscience Ultracomp eBeads (San Diego, CA), and/or ArC Amine Reactive Compensation Beads (ThermoFisher Scientific). Cells were stained following the protocol outlined above for either surface staining or Live/Dead staining. Beads were stained following manufacturer protocol.

### Flow Cytometry

Samples were analyzed with the use of BD LSRII analyzers (Becton Dickinson, Franklin Lakes, NK) configured for 18-color analysis at the University of Pennsylvania Flow Cytometry and Cell Sorting Resource Laboratory or the Stanford Shared FACS Facility and a BD LSRFortessa analyzer (Becton Dickinson, Franklin Lakes, NK) configured for 18-color analysis and a BD FACSAria III sorter configured for 13-color analysis at the PAVIR Flow Cytometry Core. CS&T beads (BD Biosciencse) were used to standardize analysis between instruments and from day to day.

Because of instrumentation change combined with improvements to the flow cytometry panels over time, some population definitions were modified. In these cases, the definition used within a figure is always specified.

### BD Rhapsody Single Cell Analysis System

Cells were thawed and stimulated with IE-1 peptide as described above for a time course for one subject, for 12 hours total without transport inhibitors added. Samples were stained with oligonucleotide-conjugated Sample Tags from the BD Human Single-Cell Multiplexing Kit in BD stain buffer following the manufacturer protocol. Barcoded samples were then washed and spun down at 350x*g* for 10 minutes and pooled. Pooled sample was then stained concurrently with oligonucleotide-conjugated antibody to CD45RA (clone HI100, sequence ID AHS0009, BD) and a panel of surface antibodies for sorting (**Supplementary Table 1D**). Stain was in BD stain buffer for 30 minutes on ice, and samples were then spun down at 350x*g* for 10 minutes and washed three times. Pellet was resuspended in Rhapsody buffer for sort and capture.

CMV-responsive (CD8^+^CD137^+^ and CD4^+^CD154^+^) T cells were sorted on a BD FACSAria III instrument in the Palo Alto Veterans Institute for Research Flow Cytometry Core set up as described (30). Cells were sorted at 4-way purity into LoBind microcentrifuge tubes (Eppendorf) in Rhapsody sample buffer with the chiller on. Cell capture and library preparation were completed using the BD Rhapsody Targeted mRNA and AbSeq Reagent Kit. Briefly, cells were captured with beads in microwell plate, followed by cell lysis, bead retrieval, cDNA synthesis, template switching and Klenow extension, and library preparation in the Stanford Human Immune Monitoring Center following the BD Rhapsody protocol (31). Libraries were prepared for T cell receptor, sample tags, targeted mRNA using the T cell panel (Human T-Cell Expression Panel) with two additional custom primer sets for genes TOX and TOX2 (panel ID 001246), and AbSeq. Sequencing was completed on NovaSeq (Illumina, San Diego, CA) in the Stanford Genome Sequencing Service Center, and at Novogene (Davis, CA).

### Data Analysis

Graphs were generated and statistics calculated in GraphPad Prism (San Diego, CA). Graphs include column plots, box and whisker plots, X-Y plots, and bar plots. Flow cytometry data were analyzed in FlowJo version 10.7.1 (BD, Ashland, OR). Unless otherwise indicated, gates were manual. Uniform Manifold Approximation and Projection [UMAP, (32)] and Flow Self-Organizing Maps [FlowSOM, (33)], analysis were conducted using FlowJo plugins. First UMAP was completed to reduce dimensions and estimate appropriate number of clusters. Then FlowSOM was run with that specified number of clusters to identify unbiased clusters in the sample.

Rhapsody data were processed using the Seven Bridges Genomics online platform (San Francisco, CA) and BD Rhapsody Targeted Analysis Pipeline with V(D)J processing incorporated. After processing, data were imported into SeqGeq version 1.6.0 (BD, Ashland, OR). The import included a CSV file of all the data, and CSV files identifying the Sample Tag and V(D)J calls. Then quality control was completed to gate out cells that were significantly smaller and with low numbers of genes expressed (dead cells), then to gate out genes expressed in fewer than 10 cells with fewer than 10 reads, or genes with expression off-scale at the upper end of expression. Then the plug-in Lex-BDSMK was run to separate out the Sample Tags, then the VDJ Explorer to identify clones. Unbiased clustering in SeqGeq was performed using the Seurat (34) plug-in. Briefly, first CD8 T cells were manually gated, then Seurat was set up to include all genes in the normal range (230 genes), to use QC to set thresholds, to log normalize, and to use UMAP as the dimensionality reduction. The output included UMAP parameters and lists of genes up and down regulated, including fold change and q-values. Further analysis was completed through manual gating.

### Statistics

Unpaired analysis of two groups was conducted through a Mann-Whitney test. Two-way comparison of two matched groups was computed using two-way analysis of variance (ANOVA) with Sidak correction. Two-way comparison of two unpaired groups was computed using mixed-effects analysis with Sidak correction for multiple comparisons. Regressions were analyzed using simple linear regression. Two-tailed testing and alpha of 0.05 were used unless otherwise noted. Statistics for each comparison are listed in figure legends.

## Results

### Telomere Length Shortens With Age and in CD57^+^ T Cells

Telomere length is commonly used to define cell age, including in immune cells (35, 36). We optimized a flow cytometry approach to quantify relative telomere length based on relative fluorescence in the FITC channel. We analyzed samples as barcoded groups (**Supplementary Figures 1A, B**). Telomere length was calculated relative to a control of the same population without telomere probe added (**Supplementary Figure 1C**). Barcoding minimized variation between measurements, as seen in analysis of replicates of healthy volunteer samples (**Supplementary Figure 1D**). In each experiment, we calculated telomere length relative to a healthy volunteer control that was present in each set.

To measure telomere length and correlate to phenotypic parameters, we analyzed relative telomere length in a cohort of 16 young (18-35 years) and old (60-80+ years) healthy individuals. CD8 protein levels based on median fluorescence intensity were statistically higher in the old, 984 (interquartile range 846.5-1263.5), than in the young (interquartile range 1113.25-1842.5, p=0.028), consistent with a previous report that CD8 expression increases with age (37). As expected, we detected shorter telomeres in CD8 T cells from older individuals (**Figure 1A**). We also detected shorter telomere length in CD57^+^ than in CD57^–^ CD8 T cells (**Figure 1B**), consistent with published work on CD57 and telomere length (38). Furthermore, the decrease in telomere length with age is specific to CD57^–^ T cells (**Figure 1B**), indicating that once a cell expresses CD57, it has become aged, with no further aging detectable using this assay.

### Telomere Length Decreases in Total CD8 T Cells Post-Transplant in a Subset of CMV^+^ and CMV^–^ Recipients

To address whether CD8 T cells that have accelerated memory inflation also exhibit accelerated aging post-transplant, we measured telomere length of CD8 T cells pre- and at various times post-transplant. First, we analyzed telomere length of total CD8 T cells, and found a trend towards decreased telomere length in the first year after transplantation in both CMV^+^ (**Figure 2A**) and CMV seronegative (CMV^–^, **Figure 2B**) subjects. This trend was statistical for two of seven CMV^+^ subjects (**Figure 2A**) and one of four CMV^–^ subjects (**Figure 2B**). There was no change in telomere length in CD57^+^ CD8 T cells (**Figures 2C, D**), consistent with the findings in **Figure 1B**. CD57^–^ cells, in contrast, had a decrease in telomere length in two CMV^+^ subjects reflecting that of the total CD8 T cell population (**Figures 2E, F**). CMV IE-1 and pp65 responsive IFNγ^+^ CD8 T cells had variable telomere length with no detected change over time (**Figure 2G**). These findings suggest that while the CD8 T cell compartment aged in some subjects during this period, CD57^+^ and IFNγ^+^ populations represent cells that have already undergone the aging process. There did not appear to be a difference in aging between the CMV^+^ and CMV^–^ subjects. However, assays of telomere length typically detect differences between groups with at least a 10 year difference in age (35, 36), and the dynamic range of this assay may not be sensitive enough to detect differences on shorter time courses. Given the strong linkage between CD57 expression and the most aged T cells (**Figure 1B**), we concluded that CD57 may be a more appropriate parameter to analyze aging in a one year period.

### T Cells From CMV^+^ Transplant Recipients Have Phenotypic Hallmarks of Aging

Using CD57 as a correlate of aging post-transplant, we compared the proportions of CD8 T cells (gated as in **Supplementary Figure 2A**) expressing CD57 in CMV^+^ (n=8-20) and CMV^–^ (n=5-7) transplant recipients. The proportion CD57^+^ of CD8 T cells is higher in CMV^+^ recipients (**Figure 3A**). When the data are compared between pre-transplant *versus* one year post-transplant, there is a trend towards an increase in CD57 in the CMV^+^ cohort (**Figure 3B**, p=0.07) and no difference in CD57 levels in the CMV^–^ cohort (**Figure 3B**, p=0.99). Induction therapy of these subjects was either rATG, which is lymphodepleting, or basiliximab, which is not (**Table 1**). In order to determine whether lymphodepletion due to induction therapy affects post-transplant aging, we compared proportion CD57^+^ of CD8 T cells in recipients with or without rATG, and found no statistical difference based on induction pre-transplant (**Figure 3C**, p=0.55) or one year post-transplant (p=0.65). Longitudinal analysis of the proportion CD57^+^ demonstrated an increase over time in CMV^+^ recipients (**Figure 3D**, p=0.004), but not in CMV^–^ recipients (**Figure 3D**, p=0.86). These data suggest a connection between the observed accelerated inflation and CD57 levels. In our original cohort, we detected memory inflation by measuring post-transplant expansion (two-fold to 20-fold expansion during the first transplant year) of polyfunctional CD8 T cells in response to stimulation with CMV IE-1 peptide (21). We defined polyfunctional cells as producing two or three of IFNγ, TNFα, and CD107a based on Boolean gating [**Supplementary Figure 2B** and (21)]. To correlate CD57 and inflation, we therefore subdivided subjects based on magnitude of inflation from baseline to one year post-transplant and plotted % CD57^+^ *versus* % polyfunctional. There was a positive correlation between % CD57^+^ and % polyfunctional specifically in those subjects with greater than two-fold inflation (**Figure 3E**, p=0.002), and not in those with a lower degree of inflation (**Figure 3E**, p=0.12). Thus, the accelerated inflation observed in CMV^+^ subjects is correlated with an increase in CD57^+^ cells post-transplant.

A key aspect of the IRP associated with CMV in the very elderly (11) is a decreasing CD4:CD8 ratio. In our transplant cohort, we found a statistical decrease in CD4:CD8 ratio within the first year after transplant specifically in CMV^+^ recipients (**Figure 3F**, p=0.007), with no statistical change in the CMV^–^ (**Figure 3F**, p=0.22). These data provide another correlate of an enhanced aging phenotype in CMV^+^ transplant recipients.

Surface expression of specific markers can identify T cell differentiation state, which has also been linked to aging. CD8 T cells that re-express CD45RA in the absence of CCR7, CD27, or CD28, termed T effector memory re-expressing CD45RA (TEMRA), are associated with terminal differentiation and commonly express CD57 (7). We found a statistical increase in the proportion of CD8 TEMRA in the CMV^+^ (**Figure 3G**, p=0.009), but not the CMV^–^ recipients from pre- to post-transplant (**Figure 3G**, p=0.43). Next, we compared frequencies of CD8 T cell subsets ranging from least to most differentiated: naïve, central memory (TCM), effector memory (TEM), and effector CD8 T cells. There were no statistical changes in any of the four populations for CMV^–^ subjects (**Figure 3H**, p>0.4). In CMV^+^ recipients, there was a statistical decrease in the naïve subset (**Figure 3H**, p=0.02) and a statistical increase in the effector population, which overlaps with the TEMRA population (**Figure 3H**, p=0.02). There were no statistical changes in TCM or TEM for CMV^+^ recipients (**Figure 3H**, p>0.3). Decreased naïve populations and expanded terminally differentiated populations are hallmarks of signatures of aging and the IRP. Thus, CD8 T cells from CMV^+^ recipients display post-transplant changes consistent with accelerated immune aging.

### Unbiased Analysis Reveals Aging of CD8 T Cells From CMV^+^ Transplant Recipients in the First Year Post-Transplant

Phenotypic analysis described above relied on biased gating for specific phenotypes. In order to conduct a less biased analysis, we completed a set of experiments using 16-parameter flow cytometry on seven CMV^+^ and four CMV^–^ subjects with time points ranging from pre-transplant to three years post-transplant. To ensure a lack of batch effect, analysis incorporated a sample from the same healthy volunteer in all experiments. We utilized UMAP dimensionality reduction (32) to analyze the compiled data from these subjects (**Figures 4A, B**). We then conducted unbiased clustering using FlowSOM (33), which identified several clusters of CD8 and CD4 T cells, as well as other cell types (**Figures 4C, D**). This analysis found seven CD57^+^ clusters (clusters 10-16), which all grouped together with unsupervised hierarchical clustering (**Figure 4D**). These CD57^+^ clusters included both CD8 (clusters 14 and 16) and CD4 (clusters 10 and 15) T cells, as well as DUMP^+^ cells (clusters 11 and 13). The DUMP^+^ are most likely CD16^+^, as cells co-expressing CD8 and CD14 or CD19 or co-expressing CD57 and CD14 or CD19 are very rare (data not shown). Thus, the DUMP^+^CD3^–^CD57^+^ cells are most likely NK cells, and the DUMP^+^CD3^+^CD8^+^ are most likely activated and highly cytotoxic T cells, including CD8^+^ NKT, but limitations in the data preclude definitive identification (39–41). We next analyzed the change in proportion of all clusters over time in the eleven patients and found that while there is variation in cluster size, the majority of clusters do not consistently change in size over time (data not shown). We next focused the analysis to the four CD57^+^ clusters which represent terminally differentiated or aged T cells. The size of the CD8^+^ CD57^+^ TEMRA cluster (cluster 14) increased over time specifically in CMV^+^ but not CMV^–^ recipients (**Figure 4E**). The CD8^+^ CD57^+^ TEMRA IFNγ^+^ TNFα^+^ CD107a^+^ population did not change consistently between subjects. In contrast, there was no CD4 T cell population that consistently increased (**Figure 4F**). Thus, both biased and unbiased approaches to analysis found a specific increase of CD8 T cells with a CD57^+^ TEMRA phenotype in CMV^+^ recipients post-transplant.

### Unbiased Analysis Reveals Changes Consistent With Post-Transplant CD8 T Cell Aging in a Subject After Transient Viremia

The analyses thus far provide evidence of aging within the first year post-transplant, but rely on a limited range of molecules and predominantly protein expression. One CMV^+^ subject, 14, was excluded from the earlier grouped analyses because of a detected episode of low level viremia (**Table 1**). To determine the impact of low level CMV reactivation on T cell immunity, we used the BD Rhapsody single cell sequencing approach on longitudinal samples from subject 14. This microwell based method allows the use of a mix of barcoded samples, inclusion of surface protein expression through staining with oligonucleotide-conjugated antibodies, and RNA sequencing for a selected panel of genes and V(D)J [**Figure 5A** and (31, 42)]. Samples were sorted as CD137^+^ after 12 hour stimulation with CMV IE-1 peptide (**Figure 5A**). We included an oligonucleotide-conjugated antibody to stain for CD45RA and used the Human T-Cell Expression Panel for gene expression. Analysis included a total of 10468 total cells, 7076 CD8 T cells, and 4494 CD8 T cells with identifiable TCR CDR3. We first measured % CD57^+^ of CD8 T cells by surface staining in this subject and found a decrease in this population from pre- to 104 days post-transplant, with a gradual increase to the end of the first year (**Figure 5B**). The increase coincided with a period immediately following an episode of low level (213 international units/mL) CMV viremia detected at day 227, as well as multiple grade 1A rejection events.

We next completed unbiased analysis of gene expression using Seurat (34), and identified eight clusters of CD8 T cells in this subject (**Figure 5C**). These clusters represented a range of expressed genes, with three dominant clusters (clusters 6-8) expressing genes associated with cytotoxicity (*PRF1*, *NKG7*, *GZMH*) and functionality (*CCL3*, *CCL5*, **Figure 5D**). Cluster 8 (cytotoxic and *HAVCR2* ^+^) increased post-transplant, in contrast to cluster 6 (cytotoxic and chemokine ligand *XCL1* ^+^), which decreased during the first 3 months followed by an increase. These patterns mirror the change in CD57 expression in **Figure 5B** (**Figures 5E, F**). Expression of cytotoxic genes (*PRF1*, *GZMH*, *NKG7*) and lack of genes associated with differentiation potential (*CCR7*, *SELL*, *IL7R*) suggests that clusters 6-8 may represent an aged phenotype (43–47). The gene expression pattern in clusters 6-8 suggests that *LAG3* and *HAVCR2* (TIM3), genes previously associated with an exhausted phenotype (48), may be implicated in immune aging as well. This analysis provides an additional correlate of post-transplant aging in CMV^+^ individuals.

### Aged and TEMRA Populations Enriched in Clonally Expanded CD8 T Cells After Transient Viremia

The above data suggest that immune aging occurs at a transcriptional level post-transplant in CMV^+^ individuals, however they do not directly identify aging cells. In the same subject, we used a set of oligo-conjugated antibody and sequenced genes previously associated with immune aging (37, 43–46, 49, 50) to identify the aged population (**Figure 6A**). This analysis found an increase in the population of aged cells post-transplant (**Figure 6B**), as well as a consistently high percentage of TEMRA (**Figure 6C**). The frequency of TEMRA decreases post-transplant, followed by an increase after 104 days (**Figure 6C**). For a control that should not increase in the context of immune aging, we also analyzed TCM, which changed in a manner inverse to that of TEMRA, with an increase after transplant followed by a decline over time (**Figure 6D**).

We took advantage of the BD Rhapsody data to address one further aspect of immune aging: that T cell clonal expansion, particularly in the context of CMV-associated memory inflation, can contribute to immune aging (10). To address this question, we used the single cell TCR sequence data. We then analyzed aging, TEMRA, and TCM phenotypes as in **Figures 6B–D** based on T cell clonality. To analyze aging relative to clone size, we grouped clones based on frequency of cells at that time point. For these analyses, we defined clones comprising <1% of CD8 T cells as representing cells that do not belong to large clonal expansions whereas those comprising >2.5% of the sample belong to clonal expansions. The percent of each clone with an aged phenotype increased post-transplant independent of clone size (**Figure 6E**). The increase occurred between days 200 and 264 for large clones, and between day 264 and day 368 for small clones (**Figure 6E**). The subject’s viremic episode was detected at day 227, indicating that the immune response to viremia included an expansion of aged large clones. TEMRA, like aged cells, expanded post-transplant independent of clone size, but represented a larger percentage of large clones at all time points (**Figure 6F**). TCM, in contrast, were most highly represented in small clones, with a decrease over time in large clones (**Figure 6G**). To compare the aging phenotype of the largest clones with others, we identified the clones that represented >5% of the sample at one or more time points (7 total clones and 1904 total cells). We measured the frequency of cells with an aged phenotype within the largest clones. Consistent with results in **Figures 6E–G**, we detected a pattern of increased aged cells within the largest clones after day 200 (**Figure 6H**), an increase in the frequency of TEMRA over the time course (**Figure 6I**), and a decrease in the frequency of TCM over the time course (**Figure 6J**). The decrease in TCM was specifically after the episode of viremia, consistent with a transition to more differentiated cells at that time. Thus, the aging phenotype observed post-transplant was enhanced in the context of clonal expansion.

## Discussion

This study addressed the hypothesis that CMV combined with transplantation leads to accelerated T cell aging. We used multiple approaches to address this hypothesis including measurement of telomere length, biased and unbiased flow cytometry phenotyping, and single cell RNA sequencing. Together, these data demonstrated that the CD8 T cell compartment accumulates an age-associated CD57^+^ subset in the first year after transplant, and that this process appears to be accelerated in CMV^+^ relative to CMV^–^ individuals. We also demonstrated that surrogate markers such as CD57 may be a more appropriate measurement of aging than telomere length in short-term longitudinal studies. Additionally, the T cell aging phenotype appears to result from relative expansion of cells belonging to a specific terminally differentiated phenotype, rather than aging within T cell subpopulations. Overall, these data provide significant insight into immune aging after transplantation.

Telomere length is the gold standard for detection of cellular aging. One study that measured changes in telomere length of bulk T cells during the first year after transplant found younger T cells one year after basiliximab but not ATG induction (51). While samples were matched for age and CMV status, the study did not specifically address longitudinal changes in individual subjects. A second study used a flow cytometry based assay for telomere length in CD4 and CD8 T cells pre-transplant, but did not assess the effect of CMV serostatus or cellular differentiation state as correlates of aging (52). Our assay was designed to evaluate the CD8^+^CMV-responsive population at a single cell rather than bulk level. Unfortunately, the variability of our telomere assay limits detection of small changes in telomere length. Development of more sensitive assays or longitudinal analysis over longer time periods may allow for more rigorous detection of telomere changes.

Our findings are largely in concordance with the previously described IRP associated with increased mortality in aged individuals (11). The IRP is characterized by increased frequency of terminally differentiated CD8 T cells, a reduced CD4:CD8 ratio, and persistent CMV infection. Chronic and end stage kidney disease are associated with the IRP, particularly in combination with CMV (15–17). CMV and immunosenescence are associated with elevated cardiovascular mortality in the elderly (14), and the inflammation associated with immunosenescence has been shown to contribute to cardiovascular disease (53). The relationship of immunosenescence to both kidney and heart disease suggests distinct implications for recipients of either organ transplant.

Previous studies of IRP in transplant recipients are also consistent with our findings. Pre-transplant IRP has been correlated with impaired post-transplant responses to infection (54). In addition, CMV seropositivity and CD57^+^CD28^–^ CD8 T cells have been correlated with cancer and heart disease after kidney transplantation (55, 56). These findings indicate that CMV and transplantation in combination could enhance the IRP, and are consistent with our findings of elevated CD57^+^ TEMRA after transplantation. One contrasting finding is that transplant induction with ATG has been associated with increased immunosenescence of T cells post-transplant (defined as accumulation of CD28^–^CD57^+^ CD8 T cells), particularly in CMV seropositive (CMV^+^) recipients (51). In our hands, induction did not appear to impact immune aging post-transplant. However, the small number of subjects limits our ability to draw conclusions on this point.

Post-transplant accelerated aging also likely impacts the T cell repertoire. Inflation of CMV-specific T cells has been shown to promote dominance of low affinity clones (57). In addition, CMV reactivation leads to a TCR repertoire with holes, or gaps in the repertoire (58). These repertoire shifts may alter responses to CMV, but also have implications for formation of other immune responses. A number of studies have investigated the impact of CMV seropositivity on responses to influenza vaccination in the immunocompetent population. CMV is associated with enhanced immunity to influenza in the young (59) and a wide range of changes in immunity in the aged [reviewed in (60)]. Thus, studies of accelerated aging in transplantation may be important to resolving the differences in data for influenza vaccine, and for study of the impacts of CMV on protective immunity against other pathogens.

These analyses, while largely targeted to highly differentiated CD8 T cells, have intriguing implications for other lymphocyte populations. For instance, the decrease in naïve T cells suggests that this population may be relevant to age-associated changes after transplantation. The naïve population could include stem cell memory T cells, a population that can generate all memory and effector T cell types (61). The unbiased analysis also identified changing populations of both CD4 T cells and NK cells, which could also be of interest for further study. CMV and aging are both known to promote variation across immune cell types between individuals (62), and studying these populations will be important to understanding the impact of this variation after transplantation.

The single cell sequencing provides a uniquely in-depth approach to understanding immunity after transplantation. By incorporating clinical data from one transplant recipient, we determined that the enhanced aging phenotype was detected after a viremic event. This finding suggests that antigen exposure contributes directly to the expansion of populations of aged phenotype, even in the setting of maintenance of immunosuppression and antiviral therapy. The corresponding clonality data further suggest that this aging process initially promotes aging of clonally expanded cells, but that cells belonging to small clones subsequently adopt an aged phenotype. TEMRA differentiation was more closely linked to clone size, with larger clones more highly represented in TEMRA, suggesting separate consideration of aged cells, TEMRA, and aged TEMRA will be appropriate. Interpretation is somewhat complicated in that expansion of aged populations is subsequent to acute rejection events, which involve a distinct form of antigen exposure.

An important question raised by this study is on what time frame accelerated aging occurs after transplantation. Many of our analyses detected change within the first year after transplantation. In **Figures 3** and **4**, data for some subjects included time points out as late as three years post-transplant. In those subjects, the accelerated aging appears to continue at the later time points. Thus, longer term analyses will be needed to assess implications for both T cell aging and for the patient.

This study has several strengths. First, the flow cytometry assay for telomere length allowed direct correlation of telomere length with the surrogate marker CD57 in an assay that is both high-throughput and measures single cells. Second, longitudinal analysis post-transplant provided a unique opportunity to study T cell aging within individuals on a relatively short time scale, providing insight into the rate at which immune aging occurred. Third, the mix of biased and unbiased analyses allowed us both to accurately define populations and to detect findings unanticipated from the biased analysis. Fourth, the targeted gene expression and TCR clonality analysis of subject 14 provided a highly in depth look at T cell immunity in one individual with a low level viremic event during the first year after transplantation. Overall, these strengths contribute to form a unique approach to provide detailed insight into post-transplant immune aging.

There are limitations that should be considered when interpreting the results of this study. First, the dynamic range and variability of the telomere assays limits the conclusions that can be drawn. Second, the relatively small number of subjects in the study limits statistical conclusions. Specifically, the number of CMV^–^ recipients is not high enough to conclusively rule out impacts in these recipients, and the small number precludes analysis subdividing the patient population based on induction therapy, donor CMV serostatus, or transplanted organ. The small sample size and relatively short time frame also limit our ability to draw conclusions about clinical outcomes. Third, we did not have absolute numbers for cell counts, so we cannot determine whether the increase of CD57^+^ TEMRA was specifically in proportion, or also in absolute number. Fourth, the BD Rhapsody data did not include direct measurement of CD57, requiring a different definition of aging cells in those analyses. We defined aging cells using other markers established in the literature resulting in a very conservative aging gate and percentages of cells lower than expected. Fifth, the population of subjects was inherently variable, representing two different organs as well as varying induction therapies (**Table 1**). Thus, further study is needed before conclusions can be drawn about the implications for any specific therapy.

Our findings overall indicate that the CD8 T cell compartment becomes more aged in the first year after transplantation in CMV^+^ individuals, with an expansion of CD57^+^ TEMRA. One major implication of these findings is that the immune alterations found in the elderly such as impaired T cell proliferation and signaling and impaired vaccine responses could impact transplant recipients as well, potentially at much younger ages than expected. Another is that aging immune populations could directly impact allograft health and survival. For instance, aged terminally differentiated cells have altered functionality, while maintaining cytokine production (7) and polyfunctionality (63). In consequence, they participate in antiviral inflammatory responses, which can be protective against ongoing exposure to CMV. On the other hand, cytokine production promotes a pro-inflammatory environment that may contribute to adverse inflammatory outcomes, such as chronic kidney rejection (64, 65). CMV exposure has been correlated with increased incidence of both cancer and atherosclerosis after kidney transplant concurrent with accumulation of CD57^+^CD28^–^ T cells (55, 56). Further, kidney transplant recipients with a high proportion CD57^+^ of CD8 T cells have elevated risk of squamous cell carcinoma (66), and kidney recipients with an elevated proportion of CD57^+^ TEMRA have an elevated risk of late graft dysfunction (67). In addition, the in-depth phenotyping suggests new approaches to study changes in T cells associated with aging. Future study will address longer term changes in immune phenotype after transplant, and correlate to clinical outcomes, including chronic T cell mediated allograft rejection.

## Data Availability Statement

Sequencing data were deposited to the Sequence Read Archive (BioProject accession number PRJNA727405 and SRA accession numbers SRR14424685 and SRR14424684).

## Ethics Statement

The studies involving human participants were reviewed and approved by Institutional Review boards at the University of Pennsylvania, Stanford University and the VA Palo Alto Health Care System. The patients/participants provided their written informed consent to participate in this study.

## Author Contributions

LEH, CEG, XJ, and MKS completed experiments. LEH, XJ, MKS, and BAP completed analyses of the data. LEH, CEG, XJ, JG, HTM, and JSM contributed to experimental design. LEH and JSM wrote the majority of the manuscript. KBM and JSM collected clinical specimens. All authors contributed to the article and approved the submitted version.

## Funding

This work was supported by awards to JSM from the American Heart Association (13IRG13640042) and the Veterans Administration (1I01CX001971) and LEH from the Stanford Translational Research and Applied Medicine Program. LEH received support from Enduring Hearts and the American Heart Association (17POST33660597) and the National Institutes of Health [T32 AI07290; K01 1K01DK123196]. CEG received support from the National Institutes of Health (K01AG068373). HTM received support from the National Institutes of Health Cooperative Centers on Human Immunology (2U19AI057229). The content is solely the responsibility of the authors and does not necessarily represent the official views of the National Institutes of Health.

## Conflict of Interest

JSM has a family member who is employed by and has an equity interest in Genentech/Roche. No patents have been filed pertaining to the results presented in this paper.

The remaining authors declare that the research was conducted in the absence of any commercial or financial relationships that could be construed as a potential conflict of interest.

## Abbreviations

CMV, Cytomegalovirus; CMV^+^, CMV seropositive; CMV^–^, CMV seronegative; TEMRA, T effector memory re-expressing CD45RA; EDTA, Ethylenediaminetetraacetic acid; DMSO, Dimethyl sulfoxide; IE-1, immediate early-1; pp65, phosphoprotein 65; CCR, C-C chemokine receptor; PE, Phycoerythrin; APC, Allophycocyanin; β2M, β2 microglobulin; PBMC, Peripheral blood mononuclear cells; TCM, T central memory; TEM, T effector memory; UMAP, Uniform Manifold Approximation and Projection; FlowSOM, Flow Self-Organizing Maps; IFNγ, Interferon γ; TNFα, tumor necrosis factor α; ANOVA, analysis of variance; CDR, complementarity determining region; IRP, Immune risk profile; rATG, rabbit anti-thymocyte globulin.
