# Supplementary material for: Association of Premature Immune Aging and Cytomegalovirus After Solid Organ Transplant
Source: Front Immunol. 2021 May 27;12:661551. doi: 10.3389/fimmu.2021.661551 (PMC8190404; doi:10.3389/fimmu.2021.661551)
Supplement: Supplementary file 1 [file Table_1.docx]

## Supplementary Figures and Tables

**Supplementary Figure 1: Gating and measurements in telomere assay:** (A) Panel of combinatorial barcoding for analysis of up to nine samples. (B) Representative gating on live cells in individual barcodes. (C) Representative gating on CD8^+^CD57^–^ and CD8^+^CD57^+^ cells (left) and telomere fluorescence (right). + probe indicates telomere probe added, and – indicates no probe control. Numbers on right of histogram are median fluorescence intensity. Relative telomere length was calculated by subtracting the – probe value from the + probe value, and demonstrated shorter telomeres in CD57^+^ (1204 – 370 = 834) than CD57^–^ (1646 – 388 = 1258) T cells. (D) Relative telomere length was calculated as in (C) with normalization to telomere length of HeLa cells within sample (high SSC-A gate in part A top left) for replicates of three healthy volunteer samples barcoded together. Lines represent medians and error bars represent interquartile range.

**Supplementary Figure 2: Gating strategies.** (A) Representative example of gating strategy on live CD8 T cells. In Figures 3 and 5, CD8 T cells were gated as depicted. In Figure 4, live cells were gated as depicted. (B) Representative gating on polyfunctional CD8 T cells. CD8 T cells from IE-1 stimulated samples were gated for expression of IFNγ, TNFα, or CD107a (top) relative to an unstimulated gating control (bottom). Boolean combinatorial gating was used to identify those cells expressing two or three of these parameters, which we defined as polyfunctional as in reference 21.

| A: Figures 1 and 2 | | |  | |  |  |
| --- | --- | --- | --- | --- | --- | --- |
| Marker | Clone | | Fluorophore | | Vendor | Surface or intracellular |
| telomere probe | N/A | | FITC | | Dako | Intracellular |
| CD57 | HNK-1 | | Pacific Blue | | BioLegend | Surface |
| Viability | N/A | | Aqua | | BioLegend | Surface |
| CD45 | HI30 | | BV605 | | BioLegend | Surface (barcode) |
| CD3 | OKT3 | | BV650 | | BioLegend | Surface |
| CD8 | RPA-T8 | | BV711 | | BioLegend | Surface |
| CD45 | HI30 | | BV785 | | BioLegend | Surface (barcode) |
| CD45 | HI30 | | BUV395 | | BD Biosciences | Surface (barcode) |
| CD45 | HI30 | | BUV496 | | BD Biosciences | Surface (barcode) |
| B2M | 2M2 | | APC | | BioLegend | Surface (barcode) |
| IFNγ | B27 | | AF700 | | Invitrogen | Intracellular |
|  |  | |  | |  |  |
| B: Figure 3 |  | |  | |  |  |
| Marker | Clone | | Fluorophore | | Vendor | Surface or intracellular |
| Viability | N/A | | Aqua | | BioLegend | Surface |
| CD3 | OKT3 | | BV650 | | BioLegend | Surface |
|  | UCHT1 | | APC Cy7 | | BioLegend | Surface |
| CD8 | RPA-T8 | | BV785 | | BioLegend | Surface |
|  |  |  | BUV496 | | BD Biosciences | Surface |
|  | HIT8a | | APC | | BD Biosciences | Surface |
| CD4 | S3.5 | | PE Cy5.5 | | Invitrogen | Surface |
|  | RPA-T4 | | BV711 | | BioLegend | Surface |
| CD57 | HNK-1 | | PE Dazzle 594 or BV570 | | BioLegend | Surface |
| CD27 | O323 | | AF700 | | BioLegend | Surface |
| CCR7 | G043H7 | | BV711 | | BioLegend | Surface |
| CD45RO | UCHL1 | | ECD | | Beckman Coulter | Surface |
|  |  |  | BUV395 | | BD Biosciences | Surface |
| TNFα | mAb11 | | FITC | | BioLegend | Intracellular |
| IFNγ | B27 | | AF700 | | Invitrogen | Intracellular |
| CD107a | H4A3 | | PE Cy7 | | BioLegend | Surface |
| CD14 | 61D3 | | PE Cy5 or BV785 | | BioLegend | Surface (Dump channel) |
| CD16 | 3G8 | | PE Cy5 or BV785 | | BioLegend | Surface (Dump channel) |
| CD19 | HIB19 | | PE Cy5 or BV785 | | BioLegend | Surface (Dump channel) |
| CD45RA | HI100 | | APC Cy7 | | BioLegend | Surface |
|  |  | |  | |  |  |
| C: Figure 4 |  | |  | |  |  |
| Marker | Clone | | Fluorophore | | Vendor | Surface or intracellular |
| TNFα | mAb11 | | FITC | | BioLegend | Intracellular |
| TIGIT | A15153G | | BV421 | | BioLegend | Surface |
| Viability | N/A | | Aqua | | BioLegend | Surface |
| PD-1 | EH12.2H7 | | BV605 | | BioLegend | Surface |
| CD3 | OKT3 | | BV650 | | BioLegend | Surface |
| CCR7 | G043H7 | | BV711 | | BioLegend | Surface |
| CD14 | 61D3 | | BV785 | | BioLegend | Surface (Dump channel) |
| CD16 | 3G8 | | BV785 | | BioLegend | Surface (Dump channel) |
| CD19 | HIB19 | | BV785 | | BioLegend | Surface (Dump channel) |
| CD45RO | UCHL1 | | BUV395 | | BD Biosciences | Surface |
| CD8 | RPA-T8 | | BUV496 | | BD Biosciences | Surface |
| TIM3 | 7D3 | | BUV737 | | BD Biosciences | Surface |
| Bcl2 | IG191E/A8 | | AF647 | | BD Biosciences | Intracellular |
| IFNγ | B27 | | AF700 | | Invitrogen | Intracellular |
| CD45RA | HI100 | | APC Cy7 | | BioLegend | Surface |
| KLRG1 | 14C2A07 | | PE | | BioLegend | Surface |
| CD57 | HNK-1 | | PE Dazzle 594 | | BioLegend | Surface |
| CD4 | S3.5 | | PE Cy5.5 | | Invitrogen | Surface |
| CD107a | H4A3 | | PE Cy7 | | BioLegend | Surface |
| D: Figure 5 and 6 | | | |  |  |  |
| Marker | | Clone | | Fluorophore | Vendor | Surface or intracellular |
| CD154 | | TRAP1 | | BV421 | BioLegend | Surface |
| Viability | | N/A | | Aqua | BioLegend | Surface |
| CD3 | | OKT3 | | BV650 | BioLegend | Surface |
| CD4 | | RPA-T4 | | BV711 | BioLegend | Surface |
| CD8 | | HIT8a | | APC | BD Biosciences | Surface |
| CD137 | | 4-1BB | | PE | BioLegend | Surface |
| CD57 | | HNK-1 | | PE Dazzle 594 | BioLegend | Surface |
| CD14 | | 61D3 | | PE Cy5 | BioLegend | Surface (Dump channel) |
| CD16 | | 3G8 | | PE Cy5 | BioLegend | Surface (Dump channel) |
| CD19 | | HIB19 | | PE Cy5 | BioLegend | Surface (Dump channel) |
| CD107a | | H4A3 | | PE Cy7 | BioLegend | Surface |

**Supplementary Table 1: Flow cytometry panels:** Panels used for flow cytometry staining in (A) Figures 1 and 2, (B) Figure 3, (C) Figure 4, and (D) Figures 5 and 6. Barcodes and dump channel are noted in parentheticals in “Surface or intracellular” column.
